# Everyday Memory in Microsurgically Treated Patients After Subarachnoid Hemorrhage

**DOI:** 10.14740/jocmr2073w

**Published:** 2015-02-09

**Authors:** Maida Koso, Kemal Dizdarevic, Jasmina Sose-Selimotic

**Affiliations:** a Department of Psychology, University of Sarajevo, Bosnia and Herzegovina; b Department of Neurosurgery, Clinical Centre and Medical School, University of Sarajevo, Bosnia and Herzegovina; c Faculty of Arts and Science, Department of Psychology, New York University, New York, USA

**Keywords:** Subarachnoid hemorrhage, Everyday memory, Failure, Perception, Motor function, Cognitive recovery

## Abstract

**Background:**

Memory declines measured by traditional tests in patients after subarachnoid hemorrhage (SAH) are well documented. Classic examinations of memory problems sometimes do not significantly correlate with memory functions in everyday life. The objective of the study was to assess the specific type of everyday memory loss in patients after microsurgical treatment of ruptured intracranial aneurysm causing SAH.

**Methods:**

The prospective controlled, randomized study was conducted using the specific tests for everyday memory measure with high ecological validity. All patients were operated on by the same neurosurgeon (KD). Preoperatively, patients were in a good grade (Hunt-Hess I or II), with no neurological deficit and no hydrocephalus postoperatively. Patients were tested at two phases: 15 and 45 days after microsurgery with the Rivermead behavioral memory test (RBMT) and the cognitive failure questionnaire (CFQ).

**Results:**

We compared the results of the tests administered in subjects that underwent microdiscectomy surgery for SAH to a control group that underwent surgery for lumbar disc herniation (DH).

**Conclusion:**

Neuropsychological assessment of operated patients who sustained SAH showed a decline, compared to the DH group, in everyday memory function. Also, we found failures in perception and motor function in operated SAH patients with a trend of cognitive recovery as time progresses.

## Introduction

Subarachnoid hemorrhage (SAH) is excess blood in the subarachnoid space, which can be caused by a large variety of pathologies [1]. In 80% of the cases, the cause of SAH is aneurysm [1-3]. The majority of patients experiencing SAH are young with a high case of fatality. In a review article, Hop et al [4] analyzed 21 studies and they found that the reported case-fatality rates varied between 32% and 67%. The average age at the time of the hemorrhage was 55 years. Patients with SAH have typical symptoms that include a sudden onset of severe headache, nausea, vomiting, neck pain, photophobia and loss of consciousness [3]. Memory and other cognitive functions are of great importance in the rehabilitation of SAH patients, and their return to normal life. Research shows that the magnitude and type of cognitive deficits depends on a large number of factors such as: if surgery is performed on patients with a ruptured or an unruptured aneurysm [5], the timing of when the operation for a ruptured cerebral aneurysm is performed [6, 7], the anatomical location of the aneurysm [8], the family, friends and medical staff support [9, 10], and the type of treatment performed, either endovascular or conventional aortic aneurysm repair [11].

Results of the Ropper and Zervas [12] study showed that 1 year after successful treatment of ruptured intracranial aneurysm 25% of patients have psychological and emotional deficits. Subsequent research showed that these problems were even more prevalent.

The research of Ogden et al [13] showed that a high proportion of subjects (89) after SAH demonstrate some mild to moderate psychosocial impairments at 10 weeks; however, some of these patients did recover some of these areas over the next 8 months. Hutter and Gilsbach [14] used a battery of cognitive tests on a sample of 31 patients with good neurological outcomes after 1 - 5 years following aneurismal SAH and early operation. They found that 28-62% patients had marked disability in the subtests of a complex choice reaction time task. Short-term memory was impaired in 53% of the patients, 21% had reduced long-term memory, and concentration was impaired in 7-16% of the SAH patients. Hutter and Gilsbach [14] concluded that a good neurological outcome does not exclude persisting neuropsychological deficits. Hutter et al [15] assessed cognitive functions in 51 patients 1 - 13 days after SAH. Their results confirmed that the severity of SAH is the most important factor related to cognitive dysfunction.

Hillis et al [5] examined two groups of patients who underwent repair of intracerebral aneurysms 3 months after surgery. In the study they examined 20 patients with unruptured aneurysm, and 27 patients who had ruptured aneurysms. Both groups performed significantly below norms on many of the neuropsychological tests after surgery. However, there were significant differences between preoperative and postoperative performance in the unruptured aneurysm groups on measures of word fluency, verbal recall, and frontal lobe function. They concluded that some of the impairments are associated with SAH, whereas others are due to general effects of neurosurgery and preoperative management.

Otawara et al [16] found that surgical repair in patients with unruptured intracranial aneurysm does not impair cognition, and cognitive dysfunction can be considered to be the result of hemorrhage, not of surgery.

Samra et al [8] reported that cognitive improvement present after 3 months, with a plateau between 9 and 15 months, was not affected by the localization of aneurysm.

Despite the results that the localization of aneurysm does not correlate with cognitive dysfunction, numerous research patients had an aneurysm in the anterior communicating artery [17-19]. Results showed very similar cognitive dysfunction in other patients with an aneurysm in other arteries.

Lloyd et al [11] examined the difference in cognitive function and quality of life in 34 patients undergoing endovascular aortic aneurysm repair and 48 patients undergoing conventional aneurysm repair, both, before and 6 months after operation. They concluded that endovascular aneurysm surgery had a similar impact on the health-related quality of life and cognitive function of patients compared with conventional aneurysm repair. Patients in both groups demonstrated significant decline in cognitive functions.

The goal of the Tuffiash et al [20] study was to identify changes in the cognitive function associated with the surgical clipping of unruptured intracerebral aneurysm. They found no evidence of subtle cognitive deficits resulting from aneurysm clipping alone, suggesting that the common impairments after surgery for ruptured aneurysms are due to SAH itself, and complications of SAH such as vasospasmor hydrocephalus or preoperative stroke.

In our study we controlled for factors such as age (there were no patients beyond 60 years of age), years of education, anesthesia, emotional and stress factors of being hospitalized (since we had a control group of patients undergoing discus hernia (DH) surgery), verbal intelligence, sex, premorbid loss of consciousness, premorbid neurological or psychiatric illness, and Hunt-Hess (HH) grade (only HH1 and HH2), and considered that all these factors can be variables that can influence the results on tests of cognitive functions. Also, to the best of our knowledge, this is the first time that the assessment of neuropsychological functions included Rivermead behavioral memory test (RBMT), which has a great ecological validity [21]. The RBMT provides a similar measure to everyday memory situations that were chosen on the basis of reported and observed memory problems of patients that sustained a head-injury [22].

The goal of our research was to assess everyday memory and cognitive failures with SAH patients 10 - 12 days after the surgery and approximately 45 days after the surgery. The results are compared to the control group of postoperative patients that underwent surgery for a DH. The control group was also hospitalized and underwent surgery and anesthesia, which makes the cognitive functioning of this group comparable to the group of SAH patients that also underwent hospitalization, surgery, and anesthesia. These two groups did not significantly differ with respect to variables: age, sex, education, premorbid health condition, and verbal intelligence.

## Method

### Participants

Two groups of patients were examined: 12 patients (seven male and five female), diagnosed with aneurismal SAH treated with surgery and 12 patients after DH surgery (five male and seven female) in two time periods.

Table 1 shows that all patients, during the time of testing, were middle-aged, with similar levels of education (level of education has been expressed by years of education) and similar level of verbal intellectual skills (measured with subtests Wechsler adult intelligence scale (WAIS) - comperhension and WAIS - similarities).

**Table 1:** Subject Characteristics in the Subarachnoid Hemorrhage (Experimental) Group and Discus Hernia (Control) Group

	SAH group (n = 12)		DH group (n = 12)		t	df	P	NS
	Mean	SD	Mean	SD				
Age	45.92	8.81	45.5	12.59	0.09	22	0.93	NS
Education	10.67	1.97	12.17	1.80	-1.95	22	0.06	NS
WAIS - comprehension	20.83	4.324	21.83	4.73	-0.54	22	0.59	NS
WAIS - similarities	19.08	2.811	20.25	3.08	-0.97	22	0.34	NS

All participants were patients at the Neurosurgory Clinic in Sarajevo where all subjects that match criteria during a 2-year period were tested. Criteria were as follows: 60 years of age or younger, no loss of consciousness longer than 10 min, good general health condition without any diagnosis of neurological or psychiatric illness in their medical history, all patients in experimental group were HH1 or HH2, good postoperative recovery, all patients were right-handed, and all were operated on by the same neurosurgeon. All patients in the experimental group had a classical surgery treatment which included craniotomy.

### Psychological assessment

The study was conducted in two phases: 15 and 45 days after the surgery.

In the first phase, we collected basic information about the patient by use of a questionnaire (name, age, medical history, data on loss of consciousness, and medications that they were using at that time). Additionally, in both phases, we administered RBMT and cognitive failure questionnaire (CFQ). Furthermore, we used WAIS - comperhension, WAIS - similarities, and WAIS - digit span for the assessment of their verbal intellectual functioning.

The RBMT was developed with the purpose to detect impairment of everyday memory functioning and to monitor changes following the treatment for memory difficulties [21]. The Swedish version of the RBMT was translated into Bosnian and parts of the test (i.e. the Story) have been adapted to the Bosnian culture. The maximum score is 24 (indicating normal memory).

CFQ is a self-report measure of failures in perception, memory, and motor function in everyday life. It contains 25 items to which the subjects respond on five-point scale (0 = never; 4 = very often). The maximum score is 100. Results on this questionnaire correlate with the results on RBMT [22].

The verbal part of Wechsler adult intelligence scale (WAIS-III [23]) was used to assess verbal intellectual functions.

## Results

### Everyday memory

Factorial analysis of variance (2 × 2) for dependent variable everyday memory showed statistically significant effects of factors group (F = 55.201; P < 0.01) and time (F = 9.412; P < 0.01), and the effect of interaction of factors time × group is also significant (F = 5.294; P = 0.031).

**Figure 1:**
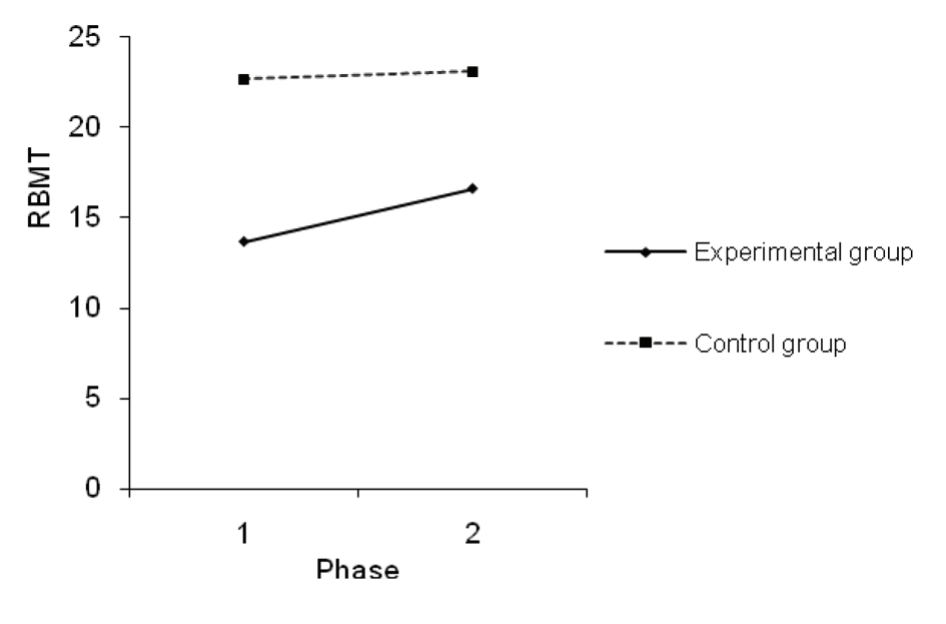
Measurement phases of everyday memory functions.

Figure 1 shows significant changes in scores on RMBT for measures in different time periods in the experimental group (ΔM_e_ = 2.91; ΔM_k_ = 0.41).

T-test for repeated measures has confirmed that this change is statistically significant for the experimental group (t = -2.929; df = 11; P = 0.014), but not for the control group (t = -0.959; df = 11; P = 0.358).

Factorial analysis of variance (2 × 2) for dependent variable of different everyday memory problems showed statistically significant effects of factor group for subtests: remembering names, remembering hidden belongings, remembering an appointment, prose recall - immediate recall, prose recall - delayed recall, face recognition, recalling a short route - immediate recall, recalling a short route - delayed recall, remembering an errand, orientation, time.

Factor time is significant for subtests: face recognition and orientation. Effect of interaction factor time × group is significant for subtest: date.

### Cognitive failures

Factorial analysis of variance (2 × 2) for dependent variable failures in perception, memory, and motor function in everyday life revealed statistically significant effects of factor group (F = 17.259, P = 0.0001) and time (F = 6.261, P = 0.020). Effect of interaction factor time × group is not significant (F = 2.231, P = 0.150).

People who had undergone aneurysm surgery, whose rupture generated SAH, show significantly higher results on CFQ test than the control group. These results indicate the presence of a greater number of occasions in which failures in perception, memory, and motor function in everyday life occur for this group of patients.

For the measurements of the first phase, participants from both groups show higher results on the CFQ test, which indicates the presence of a greater difficuly in cognitive functioning during the first phase, than was registered for the second phase (M1 = 30.165; M2 = 24.795).

**Figure 2:**
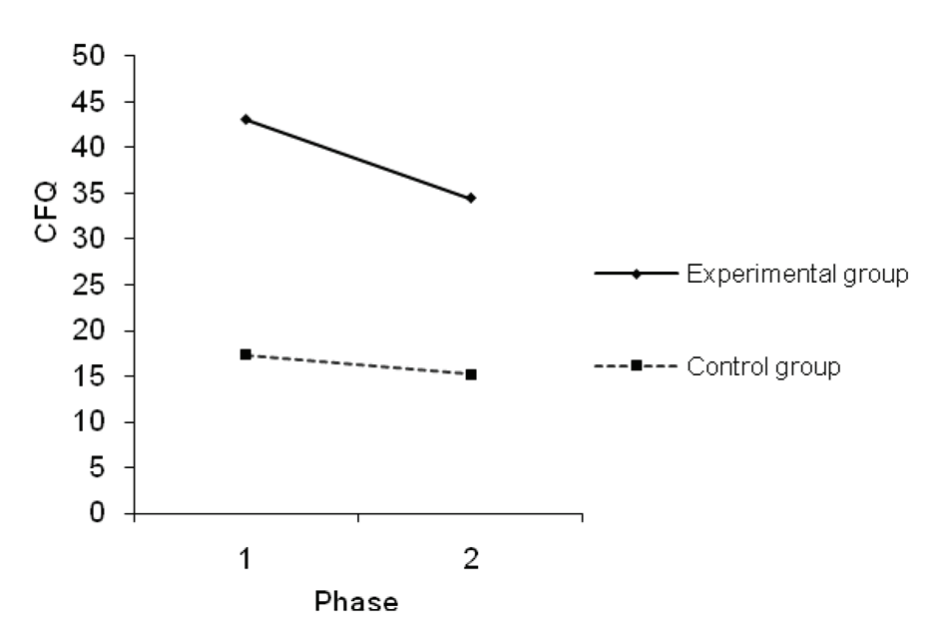
Mean values on CFQ for the first and second phases on the experimental and control groups.

As we can see in Figure 2, both groups show lower results on CFQ for the second phase, which points to the presence of a lesser number of indicators of failures in perception, memory, and motor function in everyday life (ΔM_e_ = 8.58; ΔM_k_ = 2.16). T-test for dependent means revealed that this change in results is statistically significant for the experimental group (t = 2.233; df = 11; P = 0.047), while it is below the level of statistical significance for the control group (t = 1.130; df = 11; P = 0.283).

## Discussion

Our results point to everyday memory impairment, short-term memory impairment, and problems with everyday cognitive functioning of persons who had underwent aneurysm surgery, whose rupture generated SAH.

Factorial analysis of the variance uncovered that patients show a significantly lower score on the everyday memory test (RBMT) after they have underwent aneurysm surgery (Table 2, Fig. 1). There is a significant difference bitween groups on all subtests on RBMT exept on subtest picture recognition; for most of the RBMT subtests time is not significant factor that influences difference between groups except on subtests face recognition, orientation and date; and interaction of group and time is significant for subtest date (Table 3). SAH patients also show a significantly higher score on the CFQ (Table 4).

**Table 2:** Descriptive Statistics for Everyday Memory (Maximum Result Is 24)

Group	First measurement		Second measurement	
	M	SD	M	SD
Experimental group	13.67	3.68	16.58	3.78
Control group	22.67	1.23	23.08	1.97

**Table 3:** Descriptive Statistics and Factorial Analysis of Variance (2 × 2) for Rivermead Behavioral Memory Test Subtests

RBMT subtest	Group	First phase		Second phase			Group	Time	Group × time
		M	SD	M	SD				
Remembering names	SAH	0.50	0.79	1.08	0.90	F	24.62	2.08	3.70
	DH	1.92	0.28	1.83	0.58	P η^2^	0.01 0.53	0.16 0.09	0.07 0.14
Remembering hidden belongings	SAH	1.00	0.74	0.92	0.51	F	44.62	0.46	0.01
	DH	2.00	0.01	1.92	0.29	P η^2^	0.01 0.67	0.50 0.02	1.00 0.01
Remembering an appointment	SAH	1.50	0.52	1.58	0.67	F	6.63	0.96	0.01
	DH	1.92	0.29	2.00	0.01	P η^2^	0.02 0.23	0.34 0.04	1.00 0.01
Picture recognition	SAH	1.92	0.23	1.67	0.65	F	2.51	0.51	2.05
	DH	1.92	0.29	2.00	0.01	P η^2^	0.13 0.10	0.48 0.02	0.17 0.08
Prose recall - immediate recall	SAH	0.83	0.83	0.50	0.79	F	30.94	2.39	0.86
	DH	1.92	0.29	1.83	0.39	P η^2^	0.01 0.58	0.14 0.10	0.36 0.04
Prose recall - delayed recall	SAH	1.08	0.67	0.92	0.79	F	26.40	1.00	1.00
	DH	2.00	0.01	2.00	0.01	P η^2^	0.01 0.55	0.32 0.04	0.33 0.04
Face recognition	SAH	1.33	0.78	1.83	0.39	F	0.04	19.57	0.12
	DH	1.25	0.62	1.83	0.39	P η^2^	0.01 0.53	0.01 0.47	0.74 0.01
Recalling a short route - immediate recall	SAH	1.08	0.67	1.58	0.67	F	22.00	3.67	3.67
	DH	2.00	0.01	2.00	0.01	P η^2^	0.01 0.50	0.06 0.14	0.06 0.14
Recalling a short route - delayed recall	SAH	1.00	0.74	1.42	0.79	F	30.31	1.54	1.54
	DH	2.00	0.01	2.00	0.01	P η^2^	0.01 0.58	0.23 0.06	0.23 0.06
Remembering an errand	SAH	1.58	0.79	1.83	0.58	F	5.03	0.67	0.67
	DH	2.00	0.01	2.00	0.01	P η^2^	0.03 0.19	0.42 0.03	0.42 0.03
Orientation	SAH	1.25	0.87	1.75	0.62	F	5.30	7.59	3.87
	DH	1.92	0.29	2.00	0.01	P η^2^	0.03 0.19	0.01 0.26	0.06 0.15
Date	SAH	0.58	0.67	1.50	0.80	F	13.60	4.45	13.65
	DH	1.92	0.29	1.67	0.65	P η^2^	0.01 0.38	0.05 0.17	0.01 0.38

**Table 4:** Descriptive Statistics for Failures in Perception, Memory, and Motor Function (Maximum Result Is 100)

Group	First phase		Second phase	
	M	SD	M	SD
Experimental group	43.00	16.95	34.42	17.17
Control group	17.33	9.87	15.17	11.51

Effect sizes are large and they passed the threshold of statistical significance, and are significant for both variables. As Figures 1 and 2 illustrate, as time lapses everyday memory improves as well. However, improvement of cognitive functions differs for the experimental and the control groups.

The difference between the first and second phases of measurement was not statistically significant for the control group, while the same difference reached a level of statistical significance for the experimental group. Generally we can say that after aneurysm surgery, patients have significantly lower results at both phases of the experiment. The difference in results between the first and second phases of experiment is significantly higher for the experimental group. It is evident that the experimental group shows significantly higher results in the second phase; however, those results are still lower than the results achieved by the control group participants. Patients who underwent aneurysm surgery that is induced by SAH show deficits in their everyday memory and they also show significantly higher results on the CFQ. These functions do recover with the passage of time, but that recovery is not sufficient enough to be equal to the recovery of persons who did not suffer from SAH and who did not have aneurysm surgery.

From Table 3 we can see that memory problems relate to everyday life situations. The most evident problems in the experimental group are recorded for: remembering names, remembering hidden belongings, prose recall (immediate and delayed), face recognition, and recalling a short route (immediate and delayed). Such failures usually present a serious challenge to patients that are trying to take part in their everyday duties.

The *t*-test shows that there is a statistically significant difference in the results of the subtests WAIS-digit span between the experimental and the control groups. WAIS-digit span is considered to be a measure of working memory [24], and it is used for the assessment of attention, concentration and mental control.

Our results show that memory improves with the passage of time, and this is in agreement with other research that tested cognitive functions in two or more different time periods [25, 26].

Since we managed to successfully control the influence of general anesthesia and operative stress, we can conclude that SAH and aneurysm surgery are causes of poorer performance on cognitive function tests.

Other researches that have included structural and functional brain scans could not explain or find any correlation of cognitive deficit with specific parts of the brain. Even if there was a possibility to find areas in the brain affected with a blood circulation disorder, there was no correlation between location or brain hemisphere affected with aneurysm and cognitive dysfunction [27]. The SPECT images study conducted by Tooth et al [28] identified a large common area of subcortical hypoperfusion in the SAH patient who underwent surgery. Authors of this study suggest a possible link between reduced subcortical function and the extent and severity of cognitive deficits. Nozaki et al [29] determined cholinergic dysfunction in patients with cognitive impairment after SAH on the basis of the pupillary response to tropicamide.

There is also an ongoing debate on mechanisms responsible for the recovery of cognitive functions of stroke patients. Ponsford [30] presumes that this includes different biological processes. Recovery that happens after several days is related to temporary structural damage such as vascular disruption or edema [30].

Our research also pointed out that memory dysfunction in SAH patients after aneurysm clipping will decrease over time. It is clear that the cause of memory deficit in these patients is not defined, but there is certainly some connection with previously mentioned biochemical processes in brain.

However, these and other results should emphasize the need for a neuropsychological assessment of patients after neurological surgeries so that the appropriate professional help can be provided. Many authors agree that due to such cognitive deficits it is hard for patients to return to their normal daily routine [10] while Suarez [31] believes that neuropsychological evaluation is necessary in the first 3 months after the stroke. Cognitive dysfunction symptoms that were detected in our research in people that underwent aneurysm surgery and SAH may prevent patients from going back to work, socialize, and have the same quality of life as before the surgery. Rehabilitation of SAH patients depends on our ability to recognize their problems and understand if and how cognitive deficits influence patients’ daily lives. If such deficits are permanent and appear even after rehabilitation the patient can be suggested to adapt their lifestyle and job. Also, patients with memory disorder can be included in memory rehabilitation programs. According to Wilson [32] memory rehabilitation should not be focused on the improvement of the test score for memory or any other neuropsychological functions. There are several basic steps in the memory rehabilitation process including: assessing memory and memory deficit, providing relevant information to patients and family, agreeing on therapy goals and specific problems that will be treated, choosing suitable external or internal strategies for a specific problem, teaching clients different strategies and evaluating effects of the treatment [32].

Many authors emphasize the importance of the support of community, family, and work colleagues [13].

Obviously, neuropsychological assessment and treatment have to be an essential part of any recovery process for patients with SAH including the initial phase (after aneurysm rupture and surgery). Early rehabilitation has to be available to all patients. During discharge, a rehabilitation team must inform the patient and family of future treatment, continuous cognitive and behavioral therapy, and social rehabilitation. It is also important to have an additional assessment that will enable the rehabilitation team to re-evaluate a patient’s recovery, social behavior, and gather necessary information that will help them plan all the rehabilitation stages during SAH recovery. Improvement in daily practice with patients that underwent aneurysm surgery will not be possible without a thorough research in cognitive deficit causes.
